# Estimating the healthcare cost of overweight and obesity in South Africa

**DOI:** 10.1080/16549716.2022.2045092

**Published:** 2022-04-07

**Authors:** Micheal Kofi Boachie, Evelyn Thsehla, Mustapha Immurana, Ciaran Kohli-Lynch, Karen J Hofman

**Affiliations:** a SAMRC/Wits Centre for Health Economics and Decision Science – PRICELESS SA, Faculty of Health Sciences, School of Public Health, University of the Witwatersrand, Johannesburg, South Africa; b Institute of Health Research, University of Health and Allied Sciences, Ho, Ghana; c Center for Health Services and Outcomes Research, Northwestern University, Chicago, Illinois, USA

**Keywords:** Cost, noncommunicable diseases, overweight, obesity, attributable fraction, priority setting, South Africa

## Abstract

**Background:**

Overweight and obesity are major risk factors for noncommunicable diseases. This presents a major burden to health systems and to society in South Africa. Collectively, these conditions are overwhelming public healthcare. This is happening when the country has embarked on a journey to universal health coverage, hence the need to estimate the cost of overweight and obesity.

**Objective:**

Our objective was to estimate the healthcare cost associated with treatment of weight-related conditions from the perspective of the South African public sector payer.

**Methods:**

Using a bottom-up gross costing approach, this study draws data from multiple sources to estimate the direct healthcare cost of overweight and obesity in South Africa. Population Attributable Fractions (PAF) were calculated and multiplied by each disease’s total treatment cost to apportion costs to overweight and obesity. Annual costs were estimated for 2020.

**Results:**

The total cost of overweight and obesity is estimated to be ZAR33,194 million in 2020. This represents 15.38% of government health expenditure and is equivalent to 0.67% of GDP. Annual per person cost of overweight and obesity is ZAR2,769. The overweight and obesity cost is disaggregated as follows: cancers (ZAR352 million), cardiovascular diseases (ZAR8,874 million), diabetes (ZAR19,861 million), musculoskeletal disorders (ZAR3,353 million), respiratory diseases (ZAR360 million) and digestive diseases (ZAR395 million). Sensitivity analyses show that the total overweight and obesity cost is between ZAR30,369 million and ZAR36,207 million.

**Conclusion:**

This analysis has demonstrated that overweight and obesity impose a huge financial burden on the public health care system in South Africa. It suggests an urgent need for preventive, population-level interventions to reduce overweight and obesity rates. The reduction will lower the incidence, prevalence, and healthcare spending on noncommunicable diseases.

## Background

Overweight and obesity are key threats to national and global public health in terms of prevalence, incidence, and economic burden. The conditions are caused by excessive fat in the body which results in high risk for several diseases [1]. Body Mass Index (BMI) is used as a screening tool. Individuals with BMI below 18.5 kg/m^2^ are considered underweight, those with BMI greater or equal to 30 kg/m^2^ are considered obese, while those with BMI of 25–29.9 kg/m^2^ are considered overweight [1]. Although BMI in different populations may not correspond to the same level of fat or associated health risks, it is a commonly used method of identifying at-risk individuals [1].

In South Africa, overweight and obesity are a growing public health concern. Their prevalence is rising and is currently one of the highest in sub-Saharan Africa [2]. In 2002, 56% of South African women and 29% of men were either overweight or obese. In contrast, by the end of 2016, these rates had risen to 68% of women and 31% of men [3]. One of the key contributors is an obesogenic environment characterized by the ubiquitous presence of unhealthy cheap foods, extensive marketing [4] and a fast-food industry that is growing exponentially [5]. In parallel, consumption of ultra-processed foods and sugar-sweetened beverages has grown [6], together with sedentary behavior [2,7,8].

Increased BMI is associated with higher prevalence of health complications such as cancers, cardiovascular (CVDs) and endocrine diseases, musculoskeletal and respiratory disorders [1,9–11]. The strength of the evidence on this link is provided in several systematic reviews [9,12]. Recent studies also show that overweight and obesity increase the risk of severe complications and hospitalization among COVID-19 patients [13,14]. Even before the pandemic, rising levels of overweight and obesity resulted in an increase in the prevalence of obesity-related complications. Globally these complications cause 2.8 million deaths annually [15]. In sub-Saharan Africa, disability-adjusted life years (DALYs) from NCDs increased by 67% between 1990 and 2017, placing a huge burden on a health system already struggling to deal with infectious diseases [16]. In South Africa, 9.4 million DALYs were due to NCDs [16] in 2017, and conditions such as overweight and obesity are shown to be major contributors [17,18].

A significant economic burden falls on individuals, families, and governments in the form of healthcare costs and productivity losses resulting from obesity-related conditions and their complications [19–22]. For example, direct costs range from US$0.14 million in Brazil [23] to US$23.2 trillion in South Korea [24] for obesity-related cancers. In Bangladesh, overweight and obesity cost the health system US$174 million [25]. It is estimated that 9% and 13.2% of total health expenditure in Africa and at the global level is due to overweight and obesity, respectively [26]. The associated direct cost is only a fraction of the full economic cost of overweight and obesity. However, no detailed country-specific information exists on the economic cost of overweight and obesity in sub-Saharan Africa, except for a few global projections including some African countries [26,27].

Although previous studies have estimated the healthcare cost of weight-related diseases [28–30], these studies do not quantify the cost of overweight and obesity to the public healthcare system specifically. Two studies that have attempted to quantify overweight and obesity costs do not utilize South Africa specific public sector tariffs, use methods other than gross costing, or aggregate costs for both public and private sectors [27,31,32]. A report published by one of the major private insurance administrators in 2017 shows that the healthcare cost of overweight/obesity is about R4,400 per person annually [33], although the report does not provide details on the breakdown of these costs.

Our study makes a significant contribution to the literature by estimating the healthcare cost of overweight and obesity in South Africa’s public healthcare sector based on local public sector tariffs and healthcare utilization patterns. The choice of the public sector is important because 73.8% of all healthcare utilization occurs in public health facilities, and over 80% of the population do not have medical aid [34]. The costs are estimated from a public sector payer perspective (i.e. government as the bearer/payer of the medical costs), using a time horizon of one year. Additional costs of screening and diagnosis are excluded. Consequently, the objective is to estimate the cost of overweight and obesity to South Africa’s public healthcare system and to demonstrate the cost of inaction of implementing a comprehensive NCD prevention and control strategy. This analysis also reinforces knowledge on prevalence and distribution of overweight and obesity to help design appropriate policies that address specific conditions that will be needed to inform National Health Insurance.

## Methods

### Parameters and data sources

A systematic review of the literature on the cost of obesity was conducted from January – March 2021. The goal was to identify the diseases associated with overweight and obesity as well as methodologies for costing using local evidence (see appendix 1). Parameters such as relative risk, prevalence rate, healthcare utilization rate and hospitalization rates were identified. Microsoft Office Excel (version 2111, 2021) was used for data compilation and analysis. Sources of data used in the study are summarized in appendix 2.

#### Target population

The target population was individuals seeking care in South Africa’s public healthcare sector. Using data sources such as the General Household Survey [34], Global Cancer Observatory (GLOBOCAN) [35] and 2020 mid-year population estimates, we estimated the population (≥15 years) utilizing public healthcare services [29]. Our population included the age group 15–24 years because current evidence shows that mortality from weight-related diseases such as cancer, CVDs and endocrine diseases is rising among the youth in South Africa [36].

The estimated patient-population treated in the public sector was determined as follows:

(1) PF=PRxPopxURxM

where PR is the prevalence rate of the disease, Pop is the population (≥15 years), UR is percent of sick people using healthcare, M is the percent of population (≥15 years) without medical aid, and PF is number of patients to be treated in public facilities.

Patients with private medical insurance or medical aid [34] were assumed to receive treatment in the private health sector and therefore anyone without medical aid was assumed to receive treatment from the public sector.

#### Prevalence rates

Overweight and obesity prevalence were calculated from the National Income Dynamics Study (NIDS) [37]. The NIDS is a face-to-face longitudinal survey that has tracked income, labor market participation, health, and nutrition as well as other demographic characteristics since 2008. The survey collects information from 28,000 South Africans and those living with them. These participants live in 7,300 households across South Africa [38]. NIDS is nationally representative, covering all provinces using a multistage stratified sampling technique. The survey is conducted in waves, the latest one was conducted in 2017. The interviewers of NIDS measured the height and weight of the respondents. Details on the NIDS survey, participants and methodology are provided elsewhere [38,39]. We used height and weight information from NIDS to calculate BMI by dividing weight in kilograms by the square of the height in meters, and then categorized the results according to the World Health Organization’s cut-offs [1]. Individuals with BMI 25–29.9 kg/m^2^ and ≥30 kg/m^2^ were classified as overweight and obese, respectively. This allowed us to obtain the prevalence of overweight and obesity in the South African population (≥15 years).

The 2019 General Household Survey (GHS) [34] provided prevalence rates for CVDs (except hypertension), arthritis, and asthma. The GHS is a nationally representative cross-sectional study that tracks the progress of development and identifies persistent service delivery gaps in South Africa. Some of the areas tracked by the survey are health, education, housing and energy [34]. The survey collected information from residents living in 19,649 households in 2019. Prevalence rates for diabetes and hypertension were obtained from the South African Health Review [40]. The prevalence of gallbladder/gallstones was obtained from Nyahoda [41]. Prevalence of neoplasms were derived from GLOBOCAN [35]. Prevalence rates for breast and cervical cancers were applied only to females, while that of prostate cancer was applied to the male population.

Healthcare utilization rates were estimated from the General Household Survey [34] based on the proportion of population who needed care and consulted a medical professional. Hospitalization rates were sourced from the literature (either from South Africa or international, see appendix 2) or based on assumption.

#### Relative risks for weight-related diseases

To link overweight and obesity to NCDs, we obtained from the literature the Relative Risk (RR), i.e. the risk of an event occurring relative to exposure. There are no South Africa specific RRs for weight-related diseases. Therefore, we sourced all RRs, for overweight and obesity, from previously published studies in other jurisdictions [9,12,42,43]. The RRs from these studies were reported separately for males and females, hence we used an average of the two measures as the RR for both sexes.

### Estimation of costs

One of the standard and most widely used approaches to estimating the cost of an illness is the prevalence-based approach [44,45]. This method estimates the cost of a disease based on the current number of people with the condition for a limited time period, usually a year [44,45]. This standard costing approach in health economics has been used to estimate the cost of diseases and NCD risk factors such as smoking and overweight/obesity in the literature [46–48].

#### Costing approach and the determination of healthcare costs

The per patient healthcare cost for each disease was estimated using the bottom-up gross costing approach [45], by identifying procedures used, measuring the units (in the case of medicines) and assigning a monetary value using appropriate tariffs and unit prices in the public sector. This process was used for diseases with no previous estimates and/or previous estimates provided no details on various resources or procedures used. Unless otherwise stated, all prices or cost are South Africa specific in South African Rand (ZAR).

The treatment cost for colorectal cancer was sourced from the published literature in South Africa [49]. Costs for hypertension [29,50], stroke [29,51], Ischemic Heart Disease (IHD) [29] and diabetes [28] were also derived from previous South African studies. These studies estimated the treatment cost based on resource use. South African studies show that stroke patients utilize allied services like speech-therapy and physiotherapy [50,51] hence the cost of such allied services were accounted for. For diseases like breast (chemotherapy), prostate (chemotherapy) and cervical (chemoradiotherapy) cancers, costs were estimated in line with previous studies [49,52] and clinical guidelines for the management and treatment of cancers [53–56]. Using 2013–2015 administrative data, Guzha et al. [52] estimated the cost of breast cancer treatment. This cost was estimated prior to the publication of the National Department of Health (NDOH) breast cancer treatment guideline [53], necessitating an update of this cost based on the new guidelines.

The cost of myocardial infarction was estimated based on resource use and information from previous studies [29,50]. Resources and procedures were also identified for gallstones/gallbladder [57], arthritis [58–60] and asthma [58].

Hospitalization and Out-Patient Department (OPD) consultation costs were estimated. For each disease, hospitalization cost was obtained by multiplying the average length of stay (6.1 days) [40] in 2020 by the sum of the facility and the professional fees. Even though the average length of stay on admission among diabetic patients has been estimated to be about 8 days annually [61,62], and longer for stroke patients [50], we used 6.1 days for all diseases requiring hospitalizations unless local evidence suggested otherwise. A limitation of this approach is that it assumes the same hospital days for every disease irrespective of the stage. Unless suggested by local evidence or clinical guidelines, patients were assumed to have had 2.1 consultations in 2020 [40]. The outpatient fee was multiplied by the average number of consultations to determine outpatient cost.

The final total cost per patient in 2020 was a summation of cost of medication, laboratory test, imaging, consultations, hospitalization, and specific treatment costs such as chemotherapy administration, radiation, total hip arthroplasty and laparoscopic cholecystectomy. Based on the estimated population and utilization patterns, the aggregate direct cost was estimated for each disease.

#### Cost adjustment and perspective

Tariffs or fees for various procedures and tests were taken from the public sector Uniform Patient Fee Schedule (UPFS) [63] and the National Health and Laboratory Service (NHLS) price list [64]. Cost of medicines were sourced from Medicine Price Registry (known as the Single Exit Price (SEP)) in the private sector and the tender documents of the Government of South Africa [65,66]. Public sector costs were assumed to be 70% of private sector costs. Due to data paucity for 2020, some estimates were taken from past years. Previous years costs and/or prices were adjusted for inflation using 2020 as the base year. Costs are therefore reported in South African Rand (ZAR) (2020 prices) based on Consumer Price Index (CPI) [67]. In 2020, the average ZAR-USD (US Dollar) exchange rate was ZAR15.63 [68].

#### Quantifying the burden of overweight and obesity

To quantify the direct cost attributable to overweight and obesity, we estimated Population Attributable Fractions (PAFs) for each disease using the prevalence rates of overweight and obesity and the RRs. The PAFs were calculated by adapting the formula used in previous studies [48,69] to determine the proportion of the cost of a particular disease that could actually be due to overweight or obesity. The PAFs were calculated as follows:

(2) $$PAF=\frac{p_{o}\left(RR_{disease_{i}}-1\right)}{p_{o}\left(RR_{disease_{i}}-1\right)+1}$$

Where $p_{o}$ is the prevalence rate for obesity or overweight, RR is the relative risk, $disease_{i}$ is the weight-related disease being examined and PAF is as previously defined. The PAF for each disease was then multiplied by the total estimated treatment cost of the disease to arrive at the cost of overweight and obesity (equation 3).

(3) Overweight/Obesitycost=PAFxTotalCost

### Sensitivity analysis

We conducted a sensitivity analysis by varying the parameters simultaneously. Overweight and obesity rates were varied by ±5% (relative changes) of their baseline values. This represents an absolute change of ±1.14% for overweight and ±1.34% for obesity. It was assumed that a 10% increase in the prevalence rate of obesity would increase the current prevalence rates for the diseases by 5%. The proportion of patients seeking care and hospitalization rate were also adjusted by ±5%. Given that unit cost/prices are taken from government’s published tariffs, we did not conduct sensitivity analysis for these parameters.

## Main results

**Figure 1.**
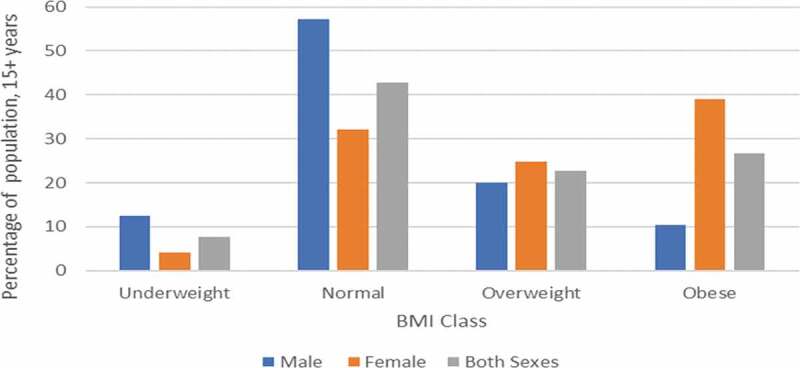
BMI classes in South Africa, 2017.

Source: Computed from NIDS [37]. Notes: Underweight: < 18.5 BMI; Normal Weight: 18.5–24.9 BMI; Overweight: 25–29.9 BMI; Obese: ≥30 BMI. Age group: ≥15 years.

Figure 1 depicts the distribution of BMI among South Africans (≥15 years) in 2017. The figure shows that 22.75% and 26.75% of adults are overweight and obese, respectively.

Table 1 shows the baseline epidemiological data of the 42.5 million South African adult population in 2020. Approximately 12 million people suffer from weight-related diseases for which treatment takes place in the public sector.

**Table 1.** Epidemiological data of obesity-related conditions at baseline

**Disease**	PR %	Patients (n)	UR %	PF (n)	Hosp. %
**Cancers**					
Breast	0.157	34,648	77	21,877	25
Prostate	0.137	29,088	77	17,736	20
Cervix	0.087	19,200	77	12,123	35
Colorectum	0.027	11,579	77	7,311	55
**CVDs/Endocrine**					
Myocardial infarction	0.850	36,1857	69	202,406	5
Diabetes	10.400	442,7425	73	2,626,812	29
Hypertension	28.200	12,005,133	69	6,715,131	5
Stroke	0.510	217,144	76	135,388	50
IHD	1.700	723,305	76	451,040	5
**Musculoskeletal Dis.**					
Arthritis	2.950	1,255,856	73	745,925	5
**Respiratory Diseases**					
Asthma	2.010	855,685	72	504,470	5
**Digestive Diseases**					
Gallbladder/gallstone	8.100	11,379,333	59	545,685	5
**All diseases**		21,077,825		11,985,905	

PR: prevalence rate; Patients: number of people suffering from the disease; UR: utilization rate; PF: number of patients treated in public facilities; Hosp: the percentage of patients treated in public facilities that were hospitalized.

This does not account for the significant number of undiagnosed diabetic and hypertensive patients and others who do not seek treatment neither does this include insurance beneficiaries who utilize services in the private sector.

Table 2 presents the relative risks (RRs) and the estimated population attributable fraction (PAF) for overweight and obesity. The PAF for breast cancer indicates that 1.79% of all breast cancer cases are attributable to overweight, while 3.36% cases are attributable to obesity. Diabetes had the highest percentage of overweight/obesity population attributable fraction, with about 29.51% from overweight and 65.57% from obesity. This was followed by arthritis with population attributable fraction of 22.55% in the overweight group and 35.75% in the obesity group.

**Table 2.** Relative risk and population attributable fraction for weight-related diseases in South Africa

Disease	Overweight		Obesity	
	RR	PAF, %	RR	PAF, %
**Cancers**				
Breast	1.080	1.790	1.130	3.360
Prostate	1.140	3.090	1.050	1.320
Cervix	1.350	7.380	1.720	16.150
Colorectum	1.480	9.840	1.810	17.720
**CVDs/Endocrine**				
Myocardial infarction	1.700	13.740	2.330	26.240
Diabetes	2.840	29.510	8.120	65.570
Hypertension	1.470	9.660	2.130	23.210
Stroke	1.190	4.140	1.500	11.800
IHD	1.300	6.390	1.670	15.200
**Musculoskeletal Dis.**				
Arthritis	2.280	22.550	3.080	35.750
**Respiratory Diseases**				
Asthma	1.230	4.970	1.610	13.930
**Digestive Diseases**				
Gallbladder/gallstone	1.270	5.790	1.880	19.050

RR: relative risk; PAF: population attributable fraction.

### Direct medical cost attributable to overweight and obesity

Table 3 shows the medical cost of treating and managing weight-related diseases in the public sector for 2020 and the fraction of the cost attributable to overweight and obesity. We estimated that ZAR352 million of the total cost of managing and treating cancers among adults are due to overweight and obesity. The main cost drivers for cancer are cervical and colorectal cancers, contributing ZAR185 million and ZAR90 million, respectively. Similarly, ZAR28,734 million of the cost of treating and managing cardiovascular and endocrine diseases were attributed to overweight and obesity, with approximately 91% of this cost coming from hypertension and diabetes. Musculoskeletal and respiratory diseases, respectively, contributed ZAR3,353 million and ZAR360 million to the total overweight and obesity cost. Overweight/obesity cost of digestive diseases was ZAR395 million.

**Table 3.** Direct Medical Costs of Treating NCDs and Obesity cost, in million rand (ZAR)

	Med.	CA/RA/LC /PH/ST	Lab.	Scan and Imaging	OPD	Hosp.	Total	Overweight	Obesity
**Cancers**									
Breast	162	239	45	197	99	91	833	15	28
Prostate	116	65	48	252	80	223	783	24	10
Cervix	49	304	69	273	55	36	787	58	127
Colorectum	183	117	-	-	-	25	325	32	58
**All cancers**	**510**	**724**	**163**	**722**	**234**	**376**	**2,729**	**129**	**223**
**CVDs/Endocrine**									
Myocardial infar.	1,840		846	-	236	32	2,954	406	775
Diabetes	10,981		3,974		1,203	4,730	20,888	6,164	13,697
Hypertension	3,380		714		13,275	2,085	19,454	1,879	4,515
Stroke	538	1,292	78	118	193	1,183	3,403	141	401
IHD	2,163		946	-	321	70	3,501	224	532
**All CVDs/Endo.**	**18,903**	**1,292**	**6,558**	**118**	**15,228**	**8,100**	**50,200**	**8,813**	**19,921**
**Musculoskeletal Disorders**									
Arthritis	880	2,603	6.38	988	1,042	232	5,750	1,297	2,056
**Respiratory Diseases**									
Asthma	638			668	442	157	1,905	95	265
**Digestive Diseases**									
Gallbladder/Gallstones	243	83	490	510	262	0.26	1,588	92	303
**All Diseases**	**21,174**	**4,702**	**7,217**	**3,006**	**17,209**	**8,864**	**62,172**	**10,426**	**22,768**

Med: medicines; CA: chemotherapy administration; RA: radiation; LC: laparoscopic cholecystectomy; PH: physiotherapy; speech-therapy; Lab: laboratory; Hosp: hospitalization All amounts have been rounded to the nearest rand.

Overall, the total cost of overweight and obesity in 2020 was ZAR33,194 million (Table 3). The disease disaggregation showed that the cost of overweight and obesity was highly prevalent in the cardiovascular and endocrine diseases category, accounting for 87% of all costs due to overweight and obesity. These costs represented 15.38% of the total government health expenditure [40] and 0.67% of GDP in 2020.

#### Disease specific healthcare cost

Healthcare cost of treating and managing weight-related diseases for approximately 12 million patients (see Table 1) amounted to ZAR62,172 million (see Table 3). The cost included medication, outpatient visits, hospitalization, scans and imaging, laboratory and other procedures associated with treatment.

Disease-specific disaggregation showed treatment costs from cardiovascular and endocrine diseases to be ZAR50,200 million. The main cost drivers in this category were hypertension and diabetes. CVDs and endocrine diseases accounted for about 81% of the total cost of managing and treating all weight-related diseases. The cost of arthritis (ZAR5,750 million) and asthma (ZAR1,905 million) were also high, accounting for about 12% of the total cost of treatment and management. The cost of treating cancers among 59,000 patients was ZAR2,729 million, with cervical and breast cancers accounting for approximately 59% of the total cost of treatment in the cancer category. The cost from gallbladder disease (gallstones) was the lowest and totaled ZAR1,588 million. The two main drivers of the total cost of treatment were medicines and consultations (OPD).

#### Results from sensitivity analysis

Table 4 presents the results from the sensitivity analysis. The overall cost of overweight and obesity was estimated to be between ZAR30,369 million and ZAR36,207 million. The lowest estimated cost for overweight was about ZAR9,452 million while that of obesity was about ZAR20,917 million (Table 4).

**Table 4.** Sensitivity analysis for cost of overweight and obesity in 2020, million rand (ZAR)

	Lower Estimate		Upper Estimate	
Cancers	Overweight	Obesity	Overweight	Obesity
Breast	13	25	17	31
Prostate	22	9	27	11
Cervix	53	116	64	139
Colorectum	29	53	35	63
**All cancers**	117	203	142	244
**CVDs/Endocrine**				
Myocardial infarction	369	709	445	845
Diabetes	5,589	12,652	6,781	14,805
Hypertension	1,697	4,106	2,075	4,954
Stroke	125	359	158	448
IHD	202	483	246	583
**All CVDs/Endocrine**	7,983	18,310	9,705	21,635
**Musculoskeletal Disorders**				
Arthritis	1,183	1,888	1,418	2,234
**Respiratory Diseases**				
Asthma	85	240	105	292
**Digestive Diseases**				
Gallbladder /gallstones	83	276	101	331
**All Disease Categories**	**9,452**	**20,917**	**11,472**	**24,735**

## Discussion

This study estimates the healthcare cost of treating 12 million individuals with weight-related conditions who utilize healthcare services in the public sector and the proportion of the cost attributable specifically to overweight and obesity.

The total overweight and obesity cost is estimated to be between ZAR30,369 million and ZAR36,207 million. Most of this cost emanates from cardiovascular and endocrine diseases, especially hypertension and diabetes. This is because these conditions are highly prevalent and BMI is highly predictive in their incidence [9,43]. Hospitalization is a major cost driver for CVD and endocrine diseases (Table 3). Since 47% of overall deaths occur in a health facility and hypertensive diseases and diabetes are among the top ten causes of death in South Africa [70], this high cost is expected. The high prevalence rates of CVDs and endocrine conditions present a huge cost to the government. Digestive diseases while significant, contributed least to the total overweight-obesity cost. Overall, about 53% of the total healthcare cost of managing and treating weight-related diseases in the public sector was attributable to the overweight and obesity problem. This cost represents approximately 15% of government health expenditure [40] and about 0.7% of GDP.

This analysis produced similar results to previous studies on overweight/obesity costs in high and middle-income countries, including Brazil [23,71], South Korea [72], Thailand [73] and Colombia [74]. The results for the cardiovascular and endocrine conditions were also in line with other studies [46,47,71,74], which showed a significant share of overweight/obesity attributed cost. The costs for other diseases were also related to estimates from other countries [25,71,74]. Using average cost estimates from Australia as a baseline [75], the World Obesity Federation projected South Africa’s cost of overweight and obesity to be US$ Purchasing Power Parity (PPP) 6,530 million in 2020 (ZAR42,000 million using PPP conversion factor 6.24) [27] similar to our upper estimate. A recent study also estimated the direct medical cost of overweight and obesity in South Africa to be US$2,460 million (about ZAR38,000 million, 0.77% of GDP) for 28 diseases in both private and public sectors [32]. A key private player in the medical scheme industry in South Africa shows that on an annual basis an obese patient incurs an extra healthcare costs of ZAR4,400 (inflation adjusted to ZAR4,951 in 2020) [33]. The per patient overweight/obesity cost of ZAR2,769 in the public sector is about 60% of the cost in the private sector.

We note, however, that direct comparison of costs estimates from different jurisdictions must be done with caution due to differences in costing methods and cost components included. Unlike our study, the Australian study also included costs of bariatric surgery and weight loss interventions among obese people, while other studies used aggregate total health expenditures [32]. In South Africa, bariatric surgery is uncommon in the public sector. Also, using total health expenditure as the basis for calculation means that costs are estimated collectively for both private and public sectors. Another issue is the fact that healthcare systems and service delivery differ across countries which makes direct comparison of studies difficult [76].

### Limitations

Our study had some limitations. Firstly, no adjustment was made for concurrent treatment of comorbidities among overweight and obese patients. Many overweight and obese people may develop more than one complication and the treatment of such complications may be simultaneous. For example, about 73% of diabetic patients develop additional NCDs for which there will be concurrent treatment [28]. Evidence from hospital records also show that a significant percentage of stroke patients also have diabetes, hypertension, cholesterol, and other heart problems. Treating or managing stroke may imply treatment for all other comorbidities [51]. Failure to account for the simultaneous treatment of comorbidities may bias the results. Secondly, the study does not consider the stage of the disease due to unavailability of data. Diseases at an advanced stage may cost more than those treated in their early stages [29]. Failure to account for such stages might underestimate the cost given that most individuals in the public sector are diagnosed late.

Thirdly, our study relied on various clinical guidelines to identify resources and clinical procedures, meaning that the degree to which clinicians adhere to these clinical guidelines could not be ascertained. This is because clinicians choose treatment modality(ies) that they deem fit based on patient-specific circumstances. For instance, surgery is one of the treatment modalities for cancer. However, we estimated the cost based on chemotherapy treatment pathway, which may underestimate the cost of cancer treatment.

Fourthly, since the main aim of this study was to show the direct costs of overweight and obesity in the public health sector, indirect costs such as productivity losses from presenteeism, absenteeism and premature mortality were excluded despite their importance. Direct costs were considered sufficient for the health sector (especially in an era of proposed National Health Insurance) which allocates resources according to the healthcare needs of the population. The estimated costs also excluded screening and diagnosis. With approximately 50% of NCDs such as diabetes being undiagnosed [28] and the data limitations outlined above, the direct cost in this study remain underestimated.

The last limitation was the lack of local data for relative risks for weight-related diseases and the exclusion of other weight-related diseases such as the corona virus disease (COVID-19). Current studies have shown that overweight and obese people are more susceptible to COVID-19 [13,14], significantly increasing costs of ICU and hospitalization [77]. Lack of detailed information on this condition therefore did not allow for such considerations. Despite these limitations, this study provides a starting point for analyzing the burden of overweight and obesity in South Africa.

### Future studies

Given our inability to ascertain the degree of compliance with the clinical guidelines, future studies counting the cost of these diseases should consider using patient records from hospitals. This will provide the exact quantity of resources and clinical procedures used in treating or managing these diseases. Future studies should also estimate to the relative risks of these diseases using South African specific data. The economic burden of overweight and obesity among COVID-19 patients is another area for further research.

## Conclusion

This study demonstrates that overweight and obesity impose significant costs on South Africa’s public healthcare system, which the country can ill-afford. The calculation is an underestimate for several reasons including the fact that screening and diagnosis are not included. We found that the cost of overweight and obesity in the public sector is ZAR33,194 (range: ZAR30,369 – ZAR36,207) million, representing about 15% of total government health spending and about 0.7% of GDP. The main drivers of the cost are cardiovascular and endocrine diseases. This is happening at a time when health budgets are being cut, when most COVID-19 deaths are attributed to underlying weight-related conditions and when people with Human Immunodeficiency Virus (HIV) are living longer. Without decisive action to prevent weight gain, the prevalence and incidence of NCDs in South Africa will continue to accelerate. If there were no overweight or obese population, Government could save about ZAR30,369 – ZAR36,207 million. A rising overweight/obese population will translate into higher healthcare cost and productivity losses from premature mortality, presenteeism and absenteeism.

The findings support the urgent need for population-level interventions to reduce overweight and obesity rates which will consequently reduce the incidence of NCDs. Global and South Africa specific evidence shows that taxation, for example, can reduce obesity rates (especially among adults), premature deaths and the associated healthcare cost through reduced consumption of sugary drinks [6,78,79]. Subsidies should be considered on healthy foods. Restrictions on marketing and advertising (including labelling) will help reduce consumption of unhealthy foods and sugary drinks linked to NCDs. Campaigns that inculcate healthy eating norms among school children will produce long-term benefits. These interventions leading to significant BMI reduction can avoid up to 2.4 million incident cases of diabetes, 1.4 to 1.7 million cardiovascular diseases, and 73,000–127,000 cases of cancer [80]. Reducing the incident cases will contribute to lower NCD healthcare costs in the longer term for South Africa. Broad population level measures to prevent cardiovascular and endocrine diseases should therefore be prioritized.

## Supplementary Material

Supplemental Material

## Patient involvement

No patients were involved.

## Paper context

The burden of noncommunicable diseases is rising globally, and overweight and obesity are major risk factors. This study comprehensively estimates the healthcare cost associated with treatment of obesity-related conditions using gross costing approach. The study finds the burden of overweight and obesity to be huge (ZAR33,194 million) hence the need for population-level interventions to reduce obesity rates in South Africa.
