# Supplementary material for: Estimating the healthcare cost of overweight and obesity in South Africa
Source: Glob Health Action. 2022 Apr 7;15(1):2045092. doi: 10.1080/16549716.2022.2045092 (PMC9004491; doi:10.1080/16549716.2022.2045092)
Supplement: Supplemental Material [file ZGHA_A_2045092_SM7491.docx]

**Title:** Estimating the healthcare cost of overweight and obesity in South Africa

**Authors**: Micheal Kofi Boachie ^1^, Evelyn Thsehla ^1^, Mustapha Immurana ^2^, Ciaran Kohli-Lynch ^1,3^, Karen Hofman ^1^,

**Supplementary material**

**Appendix 1**

**Literature review of obesity related cost-of-illness studies**

Obesity is a major cause of morbidity worldwide. This condition in which excessive fat accumulation in the body causes chronic diseases like diabetes, cardiovascular diseases (CVDs), cancers, and musculoskeletal disorders like back pain and osteoarthritis, as well as nonalcoholic steatohepatitis, and sleep apnea^1^. The prevalence of overweight or obesity has rising faster, causing 2.8 million deaths worldwide.^2^

Obesity imposes a significant economic burden on individuals, families, and countries in the form of healthcare cost and productivity losses. The healthcare cost associated with treating obesity and its direct consequences are huge. To assist policymakers in tackling the obesity problem, this paper provides a review of cost-of-illness (COI) studies on obesity related diseases. The review is a continuation of the obesity burden project.^3 4^ This review focuses on the following obesity related diseases: neoplasms (cancers), cardiovascular diseases, musculoskeletal disorders, respiratory and digestive diseases.

**Existing Reviews**

Recent systematic reviews on the economic burden of obesity show that obesity places substantial cost on countries through obesity related complications.^5-8^ Nonetheless, the heterogeneous COI methods used makes comparability of the estimates difficult. Also, none of these reviews found studies quantifying the economic costs of obesity in South Africa.

There is an extensive literature on COI studies, and all have key methodological considerations to note when estimating the burden of disease. Some of these include the type of cost (direct or indirect) to estimate and the components (e.g., hospitalizations, drugs, and ambulatory care) thereof, and the perspective (society, individual, government, or payer). Another consideration is optimal costing approach to adopt, which are usually determined, in part, by data availability. A detailed overview of various COI methods and their strengths is provided in the first part of the obesity project^4^ and elsewhere in the literature.^9-11^

**Rationale and Objectives**

The aim of this review to obtain accurate information on the cost of obesity and to inform the conceptual modelling phase of a South Africa obesity COI analysis. This is achieved through a review of cost-of-illness studies on obesity related diseases.

**Methodology**

This systematic literature review has been performed following the Preferred Reporting Items for Systematic Reviews and Meta-Analyses (PRISMA) guidelines and previous systematic reviews. This approach to literature review helps to limit bias, easily deliver required information to healthcare providers and policymakers, with reliable and accurate conclusions.^12 13^

To obtain studies on economic cost of obesity related diseases, a structured search in three databases namely PubMed, Web of Science and EconLit was conducted. The review covers studies published until December 2020. The search and review were guided by previous reviews on theme.^5-8^

*Search terms*

The following search terms were entered, and the results exported to endnote reference management software: (("Absenteeism" or "presenteeism" or “productivity” or "hospitalization costs" or “healthcare costs” or “premature mortality” or “costs and cost analysis” or “economics” or “cost benefit analysis” or “cost of illness” or “health costs” or “direct service costs” or “hospital costs” or "economics") and (“obesity” or “obesity related disease” or "metabolic syndrome" or "overweight" or "body-mass index" or "BMI")).

To be included in the review, overweight or obesity was a risk factor; (2) the estimation was based on the entire country and a representative population; (3) estimated either direct or indirect or both; (4) estimated costs were specific to obesity related diseases and provides cost estimates for each disease or group of diseases; (5) the research is in an English peer-reviewed journal; and (6) studies with any perspective in cost estimations and have attributed cost to obesity. We excluded (1) economic evaluations (cost-effectiveness, cost-utility, and cost-benefit analyses); (2) reviews, notes, commentaries, or editorials related to obesity; (4) study protocols or study designs and studies not attributing cost to obesity. For the purposes of this work, obesity is defined to include overweight.

The obesity related diseases have been grouped into five categories: neoplasms or cancers, cardiovascular diseases (includes hypertension, stroke, and ischemic heart diseases), musculoskeletal disorders, respiratory and digestive diseases.

**Results**

The literature review began on 01 January 2021 and ended on 31 March 2021 using methods outlined earlier. A summary of the studies included in the review is presented in Tables 5 and 6. Figure 1 presents the selection process of the studies. Overall, 9518 articles were retrieved. After removing duplicates and irrelevant studies, 118 articles were retained for full-text assessment. Figure 1 shows the procedures used in selecting studies.

The process resulted in 45 articles from 20 countries (Australia^14-16^, Canada^17-20^, Brazil^21-24^, United States^25-35^, Taiwan^36^, Columbia^37^, Bangladesh^38^, South Korea^39-41^, Ireland^42^, Hong Kong^43^, Germany^44-46^, New Zealand^47 48^, China^49-52^, Italy^53^, Thailand^54^, Indonesia^55^, Mexico^56^, UK^42 57^ and Switzerland^58^) as well as one cross-country study^59^ were included in the review. No study was identified for Africa. Majority of the costing studies were found in the US. The earliest study was conducted in 1992 and the latest in 2020. Tables 5 and 6 present the characteristics of and the costing methods used in the studies.

In costing obesity, majority of the studies adopt a 1-year time horizon, while some conduct their analysis for decades under different perspectives and settings. Data from budget and expenditure surveys have been the main sources for the estimation of direct medical costs of obesity-related diseases. Cost of hospitalization, ambulatory care, and drugs were some of the components included in the direct cost.^17 21^ Also, 40.3% of the studies included indirect costs of obesity in their estimations. These studies used the human capital model. Here, indirect cost is estimated based on cost of productivity losses due to premature deaths and morbidity using average daily/monthly wage data, sometimes stratified by age and sex to value days lost. In addition to the human capital approach, two studies^45 47^ quantified the indirect cost of obesity using the friction cost approach, where the initial disruption and training costs incurred by employers due to illness, injury, or premature death of the short-term period is used to value productivity losses.

Figure 1: PRISMA diagram for obesity cost-of-illness review

Records identified through database searching
(n = 9518)

Records after duplicates removed
(n = 8712)

Records screened
(n = 8712)

Records excluded based on title and abstract
(n = 8583)

Full-text articles assessed for eligibility
(n = 118)

Full-text articles excluded, with reasons
(n = 73)

- 36 studies not representative/ no specific disease cost or no monetary estimate provided.
- 9 studies focused on hypertension/diabetes alone.
- 4 studies not in English
- 5 not COI studies
- 10 review studies
- 2 reports/non peer-reviewed/conference abstracts/interviews
- 7 studies have no obesity as risk factor.

Studies included in qualitative synthesis
(n = 45)

Due to differences in setting, perspective, time-horizon or even the type of cost included, the cost estimates varied. Majority of the studies conducted their analyses from a health system perspective. In reporting, some studies, e.g., Pitayatienanan, et al. ^54^ and Anis, et al. ^17^ reported estimates in the currencies of the countries in which the studies were conducted and no US$ to local currency exchange rate was provided. Also, different currencies and inflation rates among others require a great deal of caution in comparing the estimates. For this reason, and where possible, costs have been converted and reported in 2020 US$ following established methodologies for such purpose.^60^

All studies considered one or more obesity-related diseases. We classified these diseases into five main groups: neoplasms (cancers), cardiovascular, musculoskeletal, digestive, and respiratory diseases. In South Africa, prior work ^3 4 61^ exist for diabetes and hypertension (with complications such as stroke, ischemic heart disease (IHD), and chronic kidney disease)) hence the costs associated with these cardiovascular diseases were not reviewed.

**Economic Cost of Obesity related diseases**

**1. Neoplasms and Malignant (Cancers)**

About 13 cancers have been linked to overweight and obesity. These are meningioma (cancer in the tissue covering brain and spinal cord); Adenocarcinoma of the esophagus; Multiple myeloma (cancer of blood cells); Kidneys; Uterus; Ovaries; Thyroid; Breast (post-menopausal women); Liver; Gallbladder; Upper stomach; Pancreas; Colon and rectum cancers.^62^ The reason is that fat cells (through weight gain) may release hormones that affect cell growth, leading to cancer. In the US, for example, weight-related cancers account for 40% of all cancers diagnosed.^62^ Cancer is one of the common causes of death in South Africa, accounting for over 40 000 deaths in 2017.^63^

The cost associated with obesity-related cancers have been estimated by 33 studies. The studies cover high-income countries, like Australia^15 16^, US^25 27 28 30 33^, Canada^17-19^ and the UK^57^, and upper-middle income countries such as Brazil^21 22 24^ and China^50 52^. Among studies that considered societal costs, the economic burden was highest (US$23.2 trillion) in South Korea^40^ and lowest (US$0.14 million) in Brazil^24^.

Given that breast, prostate, colorectal, and cervix cancers are among the top five cancers in South Africa, with about 140 thousand patients^64^, this study focuses on these cancers in our attempt to estimate the burden of obesity.

**2. Cardiovascular Diseases**

These are diseases affecting the artery or the heart. Common complications under this category are congestive heart failure, coronary heart disease, stroke, dyslipidemia, hyperlipidemia, pulmonary embolism, hypertension, myocardial infarction, and atrial fibrillation. We identified thirty-three studies which included one or more cardiovascular diseases in costing obesity. About 82% of the studies covered high-income countries. There was wide variation in costs given the differences in the type and components of cost included. For instance, some of the studies included indirect costs such as cost of productivity losses resulting from premature mortality and absenteeism, while others included only direct medical costs (hospitalizations, drugs, consultations, and surgeries). Sixteen studies estimated the obesity cost of cardiovascular diseases from a societal perspective. The annual cost ranged from US$0.17 million in China^51^ to US$58234 million in the United States^33^ depending on the type and composition of cost included. On the average, the burden of cardiovascular disease attributable to obesity was US$6490 million.

In 2019, 366 000 people reported having suffered myocardial infarction, majority of whom were females.^65^ These conditions were based on diagnoses from healthcare professionals. To estimate the burden of cardiovascular diseases in South Africa, and recognizing the work of Kohli-Lynch, et al. ^4^ myocardial infarction will be added to the CVDs.

**3. Musculoskeletal Disorders**

These are diseases affecting the locomotor system of the body, and are a major source of disability globally.^66^ Included in this are chronic back and neck pain, osteoarthritis, rheumatoid arthritis, psoriatic arthritis, gout, ankylosing spondylitis, osteoporosis, osteopenia, and sarcopenia. We identified 20 studies that considered one or more musculoskeletal disorders in their cost estimation. The most common complication was osteoarthritis, found in 52% of the studies considering musculoskeletal diseases. Majority (80%) of the studies on the economic burden of obesity-related musculoskeletal disorders were found in high income countries. Like the cancers, no study was found in South Africa. The average cost for the identified studies was US$3173 million, ranging from US$6.8 million in Thailand^54^ to about US$29211 million in the US^27^.

Osteoporosis and [osteo]arthritis are musculoskeletal disorders that affect 1.28 million South Africans.^65^ Therefore, our estimation of obesity cost will focus on these conditions.

**4. Respiratory Diseases**

Respiratory diseases are among the leading causes of NCD mortality worldwide, with 3.9 million deaths annually.^67^ Obesity has been identified as a risk factor for respiratory diseases such as asthma, influenza, pneumonia, exertional dyspnea, obstructive sleep apnea syndrome (OSAS), chronic obstructive pulmonary disease (COPD), obesity hypoventilation syndrome (OHS) and pulmonary fibrosis.^68 69^ Ten studies covering lower-middle^38^, upper-middle^22 37^ and high-income^17 19 20 31 41 53^ countries have estimated the economic burden of obesity related respiratory diseases. Although complications like sleep apnea, pneumonia, and chronic bronchitis were studied, asthma was the most common complication. Depending on the type of cost and the perspective, the estimated cost of obesity related respiratory diseases is US$2.72 million in South Korea^41^ and reaches US$6.2 billion in Canada^19^. The average for the group was US$906 million.

In South Africa, asthma, COPD, influenza, bronchitis and pneumonia are among the leading causes of death, accounting for about 7.1% of all deaths^63^. There are about 200 thousand people suffering from these diseases. Therefore, this study will estimate the economic burden of asthma as proxy for all respiratory diseases.

**5. Digestive diseases**

A wide range of digestive diseases, including gastroesophageal reflux disease (GERD), Barrett’s esophagus (BE), erosive esophagitis, nonalcoholic fatty liver disease (NAFLD), gallstones, pancreatitis, gallbladder, peptic ulcer, foodborne illness, and diverticular diseases are linked to obesity. These diseases impose economic cost on the healthcare system aside from their impact on productivity. Nineteen studies estimating the cost of obesity-related digestive diseases were identified. Most of the studies (74%) are in high-income countries.

Expressed in constant 2020 US$, the estimated burden of digestive diseases due to obesity ranges from US$0.72 million in Bangladesh^38^ to US$6 billion in the United States^33^, with an average of about US$1.3 billion. In 2017, disorders of pancreas, gallbladder and diseases of the stomach, duodenum and esophagus accounted for 0.7% of total mortality in South Africa.^63^ Therefore, the burden of obesity-related digestive diseases will be estimated.

**Conclusion**

There are many studies estimating the economic burden of obesity related diseases in different healthcare settings. A significant number of studies have focused on upper middle- and high-income countries, with large heterogeneity in the cost-of-illness methodology and estimates. The three methods of costing (top-down, bottom-up and econometric analysis) were found in the literature. Nonetheless, cost-of-illness methods like survey analysis and bottom-up costing were common approaches. For bottom-up costed studies, a list of important obesity complications or comorbidities were defined, and cost estimated for each complication based on resource use. The authors first measure and quantify the health inputs employed and then estimate the unit costs of the inputs used to deliver healthcare services. The top-down costing method attributes a portion of aggregate cost to obesity. Indeed, majority of the studies use population attributable fractions (PAF) to estimate the cost that can actually be attributed to obesity after obtaining the total burden of the disease. Many studies sourced direct cost estimates from survey data and health insurance claims databases. Cohort studies used subtractive approach to estimate the excess cost of obesity related diseases.

In the case of South Africa, the review found no study on the cost of obesity. Studies that estimate the total treatment and management cost exist for diabetes, hypertension, and cancers. Therefore, there is a need to estimate the economic cost of obesity focusing on direct medical cost in the public sector to inform evidence-based policymaking. In doing this, our choice of COI method was based on a bottom-up gross costing approach. The final list of diseases included will be based on data availability.

**Appendix 2**

**Methods**

The aim of this study was to estimate the healthcare cost of overweight and obesity in South Africa for the year 2020. South Africa has one of the highest prevalence of overweight and obesity in Africa. We define overweight as a body mass index (BMI) of 25 kg/m^2^ to 29.9 kg/m^2^ in adults and obesity as BMI of 30 kg/m^2^ and above. The costs are estimated for population aged 15 year or older. To estimate the cost of overweight and obesity, we use 12 obesity-related diseases: cancers (breast, prostate, cervical, and colorectum), CVDs (hypertension, stroke, myocardial infarction, ischemic heart disease), endocrine diseases (diabetes), musculoskeletal disorders (arthritis), digestive diseases (asthma) and digestive diseases (gallstones and gallbladder disease). Although there are many weight-related diseases^70^, we focus on these 12 diseases for which data is readily available.

***Sources of Data***

Following the literature review, the next step was to identify and extract relevant data for the purpose. Parameters have been identified based on the literature and data compiled from various sources (see Table 1). Acquisition of data for this study involved examination of South Africa specific open data portals including DataFirst (<https://www.datafirst.uct.ac.za/dataportal/index.php/catalog/central/about>) and Medicine Price Registry (<https://health-e.org.za/medicine-price-registry/>). The search in DataFirst produced the General Household Survey (GHS) and the National Income Dynamics Study (NIDS) from which some parameters were identified.

*General Household Survey*

The General Household Survey (GHS) is a nationally representative repeated cross-sectional study that tracks the progress of development and identifies persistent service delivery gaps in South Africa.^65^ The survey has been conducted for past 18 years. Participants are private households and residents in workers’ hostels in all South African provinces. Areas tracked include education, health, disability, social security, housing, energy, and telecommunications among others. The dataset from this survey contains information on people in 19649 households. Full details of this survey are provided in the GHS report.^65^

*National Income Dynamics Study (NIDS)*

NIDS is a longitudinal survey tracking the lives of 28000 South Africans and those living with them. These participants live in about 7300 households across South Africa.^71^ The survey has been conducted since 2008 and is conducted in waves in every two years. Five waves of NIDS have been conducted, with the recent one in 2017. Trained enumerators ask respondents questions on employment, education, health and nutrition, and many other demographic variables. In every survey, new members of the sampled household are interviewed but not followed-up. One problem associated with longitudinal surveys is attrition. Whites, Indian/Asians, and high-income respondents have had high attrition rates and therefore an extra (top-up) sample of 2,775 individuals was added. In 2019, an overview of the NIDS covering since 2008 was published.^72^ Details of the study can be found on the NIDS website: <http://www.nids.uct.ac.za/>. The sources of baseline data are presented in the Table 1 below.

Table 1: Epidemiological Information at baseline

| **Disease** | Prevalence rate (PR) | Utilisation rate (UR) | Hospitalization | Relative Risk (RR) |
| --- | --- | --- | --- | --- |
| **Overweight/obesity** | ^73^ |  |  |  |
| **Cancers** |  |  |  |  |
| Breast | ^74^ | ^65^ | ^75 76^ | ^77^ |
| Prostate | ^74^ | ^65^ | ^76 78^ | ^77^ |
| Cervix | ^74^ | ^65^ | ^76^ | ^79^ |
| Colorectum | ^74^ | ^65^ | ^76^ | ^77^ |
|  |  |  |  |  |
| **CVDs/Endocrine** |  |  |  |  |
| Myocardial infarction | ^65^ | ^65^ | Assumed | ^80^ |
| Diabetes | ^81^ | ^65^ | ^82^ | ^77^ |
| Hypertension | ^81^ | ^65^ | -do- | ^77^ |
| Stroke | ^65^ | ^65^ | -do- | ^77^ |
| IHD | ^4^ | ^65^ | -do- | ^80^ |
|  |  |  |  |  |
| **Musculoskeletal Dis.** |  |  |  |  |
| Arthritis | ^65^ | ^65^ | Assumed | ^77^ |
|  |  |  |  |  |
| **Respiratory Diseases** |  |  |  |  |
| Asthma | ^65^ | ^65^ | Assumed | ^77^ |
|  |  |  |  |  |
| **Digestive Diseases** |  |  |  |  |
| Gallbladder/gallstone | ^83^ | Assumed | Assumed | ^77 84^ |

Coverage of medical aid among adults is calculated from the General Household Survey.^65^ The parameters were used to determine the number of patients suffering from each disease and how many of them would seek treatment in public facilities. Thus, the number of patients treated in public facilities is estimated as follows:

Number of Patients treated in public facilities = PR x Pop x UR x M

Utilization rate is estimated from the General Household Survey based on a person suffering from one of the diseases consulted a health professional in the 30 days before the interview.

**Sources of cost data**

Several sources were consulted to obtain data on cost. Table 2 presents the cost and their sources.

Table 2: Sources of cost data

| Procedure/resource | Unit Cost/  Tariff (ZAR) | Source |
| --- | --- | --- |
| (Chest) X-ray | 165 | ^85^ |
| Abdominal ultrasound | 445 | ^85^ |
| Bone scan/Technetium bone scan/Gamma imaging | 445 | ^85^ |
| CT Scan chest, abdominal and pelvis | 445 | ^85^ |
| Cervical biopsy | 1770 | ^85^ |
| Bladder cystoscopy | 1770 | ^85^ |
| PET-CT scan | 12448 | ^85^ |
| MRI scan | 5490 | ^85^ |
| Spirometry test | 230 | ^85^ |
| Blood test (Complete Blood Count)/Germline blood test | 74.42 | ^86^ |
| Tumor marker test/PSA test | 225.12 | ^86^ |
| Fee for drawing blood test | 46 | ^85^ |
| Renal function test | 174.98 | ^86^ |
| CD4 count | 228.65 | ^86^ |
| Viral load | 427.26 | ^86^ |
| Kidney and liver function test | 353 | ^86^ |
| Sputum induction test | 109.20 | ^87^ |
| adioallergosorben (RAST) blood test | 96.99 | ^86^ |
| Echocardiography (test) | 1300 | ^4 61^ |
| Daily electrolytes and urea (test) | 107.37 | ^4 61^ |
| Daily blood glucose (test) | 38.20 | ^4 61^ |
| Daily lipid (test) | 130.23 | ^4 61^ |
| Daily thryroid function (test) | 403.65 | ^4 61^ |
| Beta blocker | 457 | ^61^ |
| ACE inhibitor | 1587 | ^61^ |
| Statin | 0.95 | ^4 61^ |
| OPD consultations | 170 | ^85^ |
| Hospitalisation | 1018 | ^85^ |
| laparoscopic cholecystectomy | 7598 | ^85^ |
| Physiotherapy** (Other) | 6359 (473) | ^88 89^ |
| Speech therapy | 3184 | ^88^ |
| Average non-infusion/infusion fee | 1818.50 | ^85^ |
| Total Hip Arthroplasty cost | 55650.94 | ^89^ |
| Boldocynara liver and gall bladder drops 50ml | 159 | ^90^ |
| Hypertension | 8796 | ^4 61^ |
| Stroke | 33868 | ^4 88^ |
| Diabetes | 12361 | ^3^ |
| Colorectal cancer | 48658 | ^91^ |

** per year for stroke patients; (other) refers to physiotherapy cost for arthritis patients.

**Medicine dosage and prices**

Cancer medicine use was calculated based on clinical guidelines^92-94^ and online resources for various medicine dosage^95^.

Table 3: Medicine dosage

| Drug | 5-Fluorouracil (Floracor) | Cyclophosphamide  (Endoxan Injection 500 mg) | Methotrexate | Cisplatin | Ondansetron (Cispla tab 4) | Dexamethasone (Fresenius Dexamethasone) | Olanzapine,  oral (Ranbaxy) | Docetaxel | Prednisone | Goserelin  (zoladex) |
| --- | --- | --- | --- | --- | --- | --- | --- | --- | --- | --- |
| Cycles | 6 | 6 | 6 | 6 | 6 | 6 | 6 | 2 | 2 | 2 |
| BSA | 1.73 | 1.73 | 1.73 | 1.73 | 1.73 | 1.73 | 1 | 1.73 | 1 | 1 |
| Dose (mg/m2) | 500 | 100 | 40 | 40 | 8 | 20 | 5 | 75 | 5 | 3.6 |
| No. of admin per cycle | 2 | 14 | 2 | 1 | 1 | 1 | 5 | 1 | 42 | 1 |
| Dose per admini | 865 | 173 | 69 | 69 | 14 | 35 | 5 | 130 | 5 | 4 |
| Dose per cycle in mg | 1730 | 2422 | 138 | 69 | 14 | 35 | 25 | 130 | 210 | 3.6 |
| vial/Tablet | 4 | 5 | 3 | 1 | 4 | 9 | 2 | 1 | 42 | 1 |
|  | 0 | 0 | 0 | 2 | 0 |  | 1 | 3 | 0 | 0 |
| vial/tab size | 500mg | 500mg | 50mg | 50mg, 10mg | 4mg | 4mg | 10mg,  5mg | 80mg,  20mg | 5mg | 0.01 ml (-mg injection) |
| price per tab/vial | 22.43 | 86.26 | 25.93 | 100.05 | 1.468 | 60.228 | 0.801 | 460 | 0.164286 | 1699.222 |
|  |  |  |  | 26.89 |  |  | 0.678 | 172.5 | 0 | 0 |
| Medicine cost per cycle | 89.72 | 431.3 | 77.79 | 100.05 | 5.872 | 542.052 | 1.602 | 460 | 6.9 | 1699.222 |
|  | 0 | 0 | 0 | 53.78 | 0 | 0 | 0.678 | 517.5 | 0 | 0 |
| Total medicine cost per cycle | 89.72 | 431.3 | 77.79 | 153.83 | 5.872 | 542.052 | 2.280 | 977.5 | 6.9 | 1699.222 |
| Total medicine cost X cycles (all) | 538 | 2588 | 467 | 923 | 35 | 3252 | 13.680 | 1955 | 14 | 3398 |

The study also used information from previous studies on South Africa in estimating the right dosage of medicines a cancer patient need.^96^ The medicines are prescribed in mg per body size. To calculate the dose per administration, standard body surface area (BSA) or body size is multiplied by the dose (mg/m^2^). The dose per administration is then multiplied by the number of administrations per cycle to obtain the total dose for each cycle (in the case of cancers). The dose per cycle is converted into tablets (mg) and the associated price sourced from the government tender circulars, medicine price registry or other online private pharmacies in South Africa. The cost of medicine per cycle is then calculated my multiplying the number of tablets dispensed. Table 4 shows the sources of medicine prices used in the study.

Table 4: Medicine prices

| Medicine | Price per vial/tablet (Rand) | Source |
| --- | --- | --- |
| 5-Fluorouracil (Floracor)   - 500mg | 22.43 | ^97^ |
| Cyclophosphamide (Endoxan Injection 500 mg)500mg | 86.26 | ^97^ |
| Methotrexate (Abitrexate 50)   - 50mg | 25.93 | ^97^ |
| Cisplatin (Cisacor 50mg)   - 50mg - 10mg | 100.05  26.89 | ^97^ |
| Ondansetron (Ondansetron Cipla)   - 4mg | 1.47 | ^97^ |
| Dexamethasone (Fresenius Dexamethasone)   - 4mg | 60.23 | SEP |
| Olanzapine, oral (Redilanz 5)   - 10mg - 5mg | 0.80  0.68 | ^98^ |
| Docetaxel   - 80mg - 20mg | 460  172.5 | ^97^ |
| Prednisone   - 5mg - 50mg | 0.16  4.09 | ^98^  SEP |
| Goserelin   - 0.01 ml (-mg injection) | 1699.22 | SEP |
| Morphine | 2.73 | ^4^ |
| Aspirin | 0.39 | ^4^ |
| Prochlorperazine | 167.53 | ^4^ |
| Streptokinase | 3471.13 | ^4^ |
| Enoxaparin | 19.38 | ^4^ |
| Clopidogrel | 933.39 | ^4^ |
| Paracetamol 500mg | 0.10 | ^98^ |
| methyl salicylate ointment/wintergreen | 58.58 | ^99^ |
| ibuprofen 400mg | 0.28 | ^98^ |
| Hydrochlorothiazide 12.5mg | 0.14 | ^4 98^ |
| Hydrochlorothiazide 25mg | 0.12 | ^4 98^ |
| Enalapril 10mg | 0.26 | ^4 98^ |
| Enalapril 20mg | 0.23 | ^4 98^ |
| Amlodipine 5mg | 0.12 | ^4 98^ |
| Amlodipine 10mg | 0.16 | ^4 98^ |
| Spironolactone 25mg | 0.46 | ^4 98^ |
| salbutamol (Ventimax CFC free) | 20.13 | SEP |
| influenza vaccine | 80.50 | ^100^ |
| budesonide | 192.37 | SEP |

SEP: Single Exit Price (<https://health-e.org.za/medicine-price-registry/>).

In the case of other diseases, previous studies^4 61^ and adult primary care guideline^101^ were used to determine the quantity used. Medicine prices were obtained from South African studies^4 61^, government tender circulars^97 98^ and the medicine price registry (for the private sector) where no public sector price exist. We assume that public sector price is 70% of the prices in the private sector.

**Estimating the cost attributable to overweight and obesity**

The cost attributable to overweight and obesity is estimated based on the estimated treatment cost and the Population attributable Fraction (PAF)^21^:

Overweight/obesity cost = Total cost of treatment X PAF

Total cost of treatment = Cost of (Medication + Laboratory + Imaging + OPD consultations + Hospitalizations + Other Procedures).

The cost of other procedures include radiation, total hip arthroplasty, cholecystectomies, and speech therapies.

$PAF=\frac{p_{o}({RR}_{{disease}_{i}}-1)}{p_{o}\left( {RR}_{{disease}_{i}}-1 \right)+1}$ … 1

Where $p_{o}$ is the prevalence rate for obesity or overweight, RR is the relative risk, disease i is the obesity related disease being examined and PAF is as previously defined. The calculation is performed separately for overweight and obesity. The prevalence rates of overweight and obesity are presented in Figure 1 in the main manuscript.

Other information or assumptions.

1. Only a fraction of people needing care will utilize healthcare services. This is based on the healthcare utilization patterns.^65^
2. The public health sector serves over 80% of South Africans, or those without medical aid. ^65^
3. A 10% increase in obesity rate is expected to increase the prevalence rate of various diseases by 5%. This is based on the link between obesity and NCDs, thus prevalence of NCDs increases as obesity/overweight rate rises.
4. All patients with stroke will utilize physiotherapy and speech therapy.^4 61 88 102^
5. Medicine prices and procedure tariffs are sourced from Tender documents of the government of South Africa, Medicine Price Registry (Single Exit Price) and UPFS 2020. Whenever appropriate, 70% of private sector tariff has been applied.
6. In the case of gallstones, only 33% of all cases are symptomatic and this formed the basis for cost calculation.^83^ We assumed that 2% of patients will use cholecystectomy as treatment procedure.
7. All arthritis patients were assumed to use the services of physiotherapist. Although the guideline does not prescribe arthroplasty, we found that some patients undergo treatment using this procedure.^89^ Therefore, 0.1% of all arthritis patients were assumed to use arthroplasty procedures. We also assumed that only 0.5% of all patients will do laboratory test.
8. In cases where the cost in published literature has no consultation or hospitalisation, an adjustment was made using current tariffs.

**References**

1. Centre for Diseases Control. The Health Effects of Overweight and Obesity.: CDC; 2020 [updated Sep 17, 2020. Available from: <https://www.cdc.gov/healthyweight/effects/index.html2021>.

2. World Health Organisation. Obesity. Geneva: WHO,; 2020 [Available from: <https://www.who.int/news-room/facts-in-pictures/detail/6-facts-on-obesity#:~:text=Obesity%20has%20reached%20epidemic%20proportions,%2D%20and%20middle%2Dincome%20countries>. accessed 31 January 2021.

3. Erzse A, Stacey N, Chola L, et al. The direct medical cost of type 2 diabetes mellitus in South Africa: a cost of illness study. *Global Health Action* 2019;12(1):1636611.

4. Kohli-Lynch CN, Erzse A, Hofman K. Hypertension in South Africa: Health and Economic Burden of Disease: PRICELESS SA, 2020.

5. Tremmel M, Gerdtham UG, Nilsson PM, et al. Economic Burden of Obesity: A Systematic Literature Review. *Int J Environ Res Public Health* 2017;14(4) doi: 10.3390/ijerph14040435 [published Online First: 2017/04/20]

6. Specchia ML, Veneziano MA, Cadeddu C, et al. Economic impact of adult obesity on health systems: a systematic review. *Eur J Public Health* 2015;25(2):255-62. doi: 10.1093/eurpub/cku170 [published Online First: 2014/10/17]

7. Withrow D, Alter DA. The economic burden of obesity worldwide: a systematic review of the direct costs of obesity. *Obes Rev* 2011;12(2):131-41. doi: 10.1111/j.1467-789X.2009.00712.x [published Online First: 2010/02/04]

8. Dee A, Kearns K, O'Neill C, et al. The direct and indirect costs of both overweight and obesity: a systematic review. *BMC Res Notes* 2014;7:242. doi: 10.1186/1756-0500-7-242 [published Online First: 2014/04/18]

9. Jo C. Cost-of-illness studies: concepts, scopes, and methods. *Clinical and Molecular Hepatology* 2014;20(4):327.

10. Chapko MK, Liu CF, Perkins M, et al. Equivalence of two healthcare costing methods: bottom‐up and top‐down. *Health economics* 2009;18(10):1188-201.

11. Hendriks ME, Kundu P, Boers AC, et al. Step-by-step guideline for disease-specific costing studies in low-and middle-income countries: a mixed methodology. *Global Health Action* 2014;7(1):23573.

12. Gopalakrishnan S, Ganeshkumar P. Systematic reviews and meta-analysis: understanding the best evidence in primary healthcare. *Journal of family medicine and primary care* 2013;2(1):9.

13. Greenhalgh T. Papers that summarise other papers (systematic reviews and meta-analyses). *Bmj* 1997;13(315)

14. Ackerman IN, Bohensky MA, Zomer E, et al. The projected burden of primary total knee and hip replacement for osteoarthritis in Australia to the year 2030. *BMC Musculoskelet Disord* 2019;20(1):90. doi: 10.1186/s12891-019-2411-9 [published Online First: 2019/02/25]

15. Aitken RJ, Allman-Farinelli MA, King LA, et al. Current and future costs of cancer, heart disease and stroke attributable to obesity in Australia - a comparison of two birth cohorts. *Asia Pacific Journal of Clinical Nutrition* 2009;18(1):63-70.

16. Segal L, Carter R, Zimmet P. THE COST OF OBESITY - THE AUSTRALIAN PERSPECTIVE. *Pharmacoeconomics* 1994;5:45-52. doi: 10.2165/00019053-199400051-00009

17. Anis AH, Zhang W, Bansback N, et al. Obesity and overweight in Canada: an updated cost-of-illness study. *Obes Rev* 2010;11(1):31-40. doi: 10.1111/j.1467-789X.2009.00579.x [published Online First: 2009/05/06]

18. Birmingham CL, Muller JL, Palepu A, et al. The cost of obesity in Canada. *Cmaj* 1999;160(4):483-8. [published Online First: 1999/03/19]

19. Krueger H, Krueger J, Koot J. Variation across Canada in the economic burden attributable to excess weight, tobacco smoking and physical inactivity. *Can J Public Health* 2015;106(4):e171-7. doi: 10.17269/cjph.106.4994 [published Online First: 2015/08/19]

20. Moffatt E, Shack LG, Petz GJ, et al. The cost of obesity and overweight in 2005: a case study of Alberta, Canada. *Can J Public Health* 2011;102(2):144-8. doi: 10.1007/bf03404164 [published Online First: 2011/05/26]

21. Bahia L, Coutinho ES, Barufaldi LA, et al. The costs of overweight and obesity-related diseases in the Brazilian public health system: cross-sectional study. *BMC Public Health* 2012;12:440. doi: 10.1186/1471-2458-12-440 [published Online First: 2012/06/21]

22. de Oliveira ML, Santos LMP, da Silva EN. Direct Healthcare Cost of Obesity in Brazil: An Application of the Cost-of-Illness Method from the Perspective of the Public Health System in 2011. *Plos One* 2015;10(4) doi: 10.1371/journal.pone.0121160

23. Rtveladze K, Marsh T, Webber L, et al. Health and economic burden of obesity in Brazil. *PLoS One* 2013;8(7):e68785. doi: 10.1371/journal.pone.0068785 [published Online First: 2013/07/23]

24. Sichieri R, do Nascimento S, Coutinho W. The burden of hospitalization due to overweight and obesity in Brazil. *Cad Saude Publica* 2007;23(7):1721-7. doi: 10.1590/s0102-311x2007000700025 [published Online First: 2007/06/19]

25. Hong YR, Huo J, Desai R, et al. Excess Costs and Economic Burden of Obesity-Related Cancers in the United States. *Value Health* 2019;22(12):1378-86. doi: 10.1016/j.jval.2019.07.004 [published Online First: 2019/12/07]

26. Wolf AM, Colditz GA. Social and economic effects of body weight in the United States. *Am J Clin Nutr* 1996;63(3 Suppl):466s-69s. doi: 10.1093/ajcn/63.3.466 [published Online First: 1996/03/01]

27. Wolf AM, Colditz GA. Current estimates of the economic cost of obesity in the United States. *Obes Res* 1998;6(2):97-106. doi: 10.1002/j.1550-8528.1998.tb00322.x [published Online First: 1998/04/17]

28. Colditz GA. Economic costs of obesity. *Am J Clin Nutr* 1992;55(2 Suppl):503s-07s. doi: 10.1093/ajcn/55.2.503s [published Online First: 1992/02/01]

29. Lightwood J, Bibbins-Domingo K, Coxson P, et al. Forecasting the future economic burden of current adolescent overweight: an estimate of the coronary heart disease policy model. *Am J Public Health* 2009;99(12):2230-7. doi: 10.2105/ajph.2008.152595 [published Online First: 2009/10/17]

30. Oster G, Edelsberg J, O'Sullivan AK, et al. The clinical and economic burden of obesity in a managed care setting. *Am J Manag Care* 2000;6(6):681-9. [published Online First: 2000/09/08]

31. Pearlman DN, Kaw D, O'Connell S, et al. The economic burden of preventable chronic diseases in Rhode Island. *R I Med J (2013)* 2014;97(8):36-9. [published Online First: 2014/08/02]

32. Thompson D, Edelsberg J, Kinsey KL, et al. Estimated economic costs of obesity to U.S. business. *Am J Health Promot* 1998;13(2):120-7. doi: 10.4278/0890-1171-13.2.120 [published Online First: 1999/05/27]

33. Wolf AM, Colditz GA. THE COST OF OBESITY - THE UNITED-STATES PERSPECTIVE. *Pharmacoeconomics* 1994;5:34-37. doi: 10.2165/00019053-199400051-00007

34. Nguyen NH, Khera R, Ohno-Machado L, et al. Annual Burden and Costs of Hospitalization for High-Need, High-Cost Patients With Chronic Gastrointestinal and Liver Diseases. *Clinical Gastroenterology and Hepatology* 2018;16(8):1284-+. doi: 10.1016/j.cgh.2018.02.015

35. Wang G, Zheng ZJ, Heath G, et al. Economic burden of cardiovascular disease associated with excess body weight in U.S. adults. *Am J Prev Med* 2002;23(1):1-6. doi: 10.1016/s0749-3797(02)00448-8 [published Online First: 2002/07/03]

36. Fu T, Wen T, Yeh P, et al. Costs of metabolic syndrome-related diseases induced by obesity in Taiwan. *Obes Rev* 2008;9 Suppl 1:68-73. doi: 10.1111/j.1467-789X.2007.00441.x [published Online First: 2008/03/01]

37. Gil-Rojas Y, Garzón A, Hernández F, et al. Burden of Disease Attributable to Obesity and Overweight in Colombia. *Value Health Reg Issues* 2019;20:66-72. doi: 10.1016/j.vhri.2019.02.001 [published Online First: 2019/04/30]

38. Hoque ME, Molla AA, Hoque DME, et al. Health care cost of overweight-related diseases in Bangladesh. *Public Health Nutrition* 2020;23(13):2395-401. doi: 10.1017/s1368980020001068

39. Kang JH, Jeong BG, Cho YG, et al. Socioeconomic costs of overweight and obesity in Korean adults. *J Korean Med Sci* 2011;26(12):1533-40. doi: 10.3346/jkms.2011.26.12.1533 [published Online First: 2011/12/08]

40. Lee JE, Nam CM, Lee SG, et al. The economic burden of cancer attributable to obesity in Korea: A population-based cohort study. *Eur J Cancer Care (Engl)* 2019;28(5):e13084. doi: 10.1111/ecc.13084 [published Online First: 2019/05/16]

41. Lee JW, Choi YE, Kim DW, et al. Trends in socioeconomic costs of morbid obesity among Korean adults, 2009-2013: Data from National Health Insurance Service. *Obes Res Clin Pract* 2018;12(4):389-93. doi: 10.1016/j.orcp.2017.04.010 [published Online First: 2017/05/20]

42. Keaver L, Webber L, Dee A, et al. Application of the UK foresight obesity model in Ireland: the health and economic consequences of projected obesity trends in Ireland. *PLoS One* 2013;8(11):e79827. doi: 10.1371/journal.pone.0079827 [published Online First: 2013/11/16]

43. Ko GT. The cost of obesity in Hong Kong. *Obes Rev* 2008;9 Suppl 1:74-7. doi: 10.1111/j.1467-789X.2007.00442.x [published Online First: 2008/03/01]

44. Konnopka A, Bodemann M, Konig HH. Health Burden and Costs of Obesity and Overweight in Germany. *European Journal of Health Economics* 2011;12(4):345-52.

45. Lehnert T, Streltchenia P, Konnopka A, et al. Health Burden and Costs of Obesity and Overweight in Germany: An Update. *European Journal of Health Economics* 2015;16(9):957-67.

46. Sander B, Bergemann R. Economic Burden of Obesity and Its Complications in Germany. *European Journal of Health Economics* 2003;4(4):248-53.

47. Lal A, Moodie M, Ashton T, et al. Health care and lost productivity costs of overweight and obesity in New Zealand. *Aust N Z J Public Health* 2012;36(6):550-6. doi: 10.1111/j.1753-6405.2012.00931.x [published Online First: 2012/12/12]

48. Swinburn B, Ashton T, Gillespie J, et al. Health care costs of obesity in New Zealand. *Int J Obes Relat Metab Disord* 1997;21(10):891-6. doi: 10.1038/sj.ijo.0800486 [published Online First: 1997/11/05]

49. Li Q, Cai L, Cui W, et al. Economic burden of obesity and four obesity-related chronic diseases in rural Yunnan Province, China. *Public Health* 2018;164:91-98. doi: 10.1016/j.puhe.2018.07.024 [published Online First: 2018/09/18]

50. Zhang J, Chaaban J. The economic cost of physical inactivity in China. *Prev Med* 2013;56(1):75-8. doi: 10.1016/j.ypmed.2012.11.010 [published Online First: 2012/12/04]

51. Zhao W, Zhai Y, Hu J, et al. Economic burden of obesity-related chronic diseases in Mainland China. *Obes Rev* 2008;9 Suppl 1:62-7. doi: 10.1111/j.1467-789X.2007.00440.x [published Online First: 2008/03/01]

52. Popkin BM, Kim S, Rusev ER, et al. Measuring the full economic costs of diet, physical activity and obesity-related chronic diseases. *Obes Rev* 2006;7(3):271-93. doi: 10.1111/j.1467-789X.2006.00230.x [published Online First: 2006/07/27]

53. Migliore E, Pagano E, Mirabelli D, et al. Hospitalization rates and cost in severe or complicated obesity: an Italian cohort study. *BMC Public Health* 2013;13:544. doi: 10.1186/1471-2458-13-544 [published Online First: 2013/06/07]

54. Pitayatienanan P, Butchon R, Yothasamut J, et al. Economic costs of obesity in Thailand: a retrospective cost-of-illness study. *BMC Health Serv Res* 2014;14:146. doi: 10.1186/1472-6963-14-146 [published Online First: 2014/04/03]

55. Riantoro BD, Kristina SA, Endarti D. Estimating Premature Mortality Cost of Cancers Attributable to Obesity in Indonesia. *Asian Pac J Cancer Prev* 2019;20(1):87-90. doi: 10.31557/apjcp.2019.20.1.87 [published Online First: 2019/01/27]

56. Rtveladze K, Marsh T, Barquera S, et al. Obesity prevalence in Mexico: impact on health and economic burden. *Public Health Nutr* 2014;17(1):233-9. doi: 10.1017/s1368980013000086 [published Online First: 2013/02/02]

57. Scarborough P, Bhatnagar P, Wickramasinghe KK, et al. The economic burden of ill health due to diet, physical inactivity, smoking, alcohol and obesity in the UK: an update to 2006-07 NHS costs. *J Public Health (Oxf)* 2011;33(4):527-35. doi: 10.1093/pubmed/fdr033 [published Online First: 2011/05/13]

58. Schmid A, Schneider H, Golay A, et al. Economic burden of obesity and its comorbidities in Switzerland. *Soz Praventivmed* 2005;50(2):87-94. doi: 10.1007/s00038-004-4067-x [published Online First: 2005/05/20]

59. Lette M, Bemelmans WJE, Breda J, et al. Health Care Costs Attributable to Overweight Calculated in a Standardized Way for Three European Countries. *European Journal of Health Economics* 2016;17(1):61-69.

60. Turner HC, Lauer JA, Tran BX, et al. Adjusting for Inflation and Currency Changes Within Health Economic Studies. *Value in Health* 2019;22(9):1026–32. doi: 10.1016/j.jval.2019.03.021

61. Basu S, Wagner RG, Sewpaul R, et al. Implications of scaling up cardiovascular disease treatment in South Africa: a microsimulation and cost-effectiveness analysis. *The Lancet Global Health* 2019;7(2):e270-e80.

62. Centre for Diseases Control. Cancers Associated with Overweight and Obesity Make up 40 percent of Cancers Diagnosed in the United States. 2017 [updated 03 October 2017. Available from: <https://www.cdc.gov/media/releases/2017/p1003-vs-cancer-obesity.html#:~:text=The%20International%20Agency%20for%20Research,%2C%20uterus%2C%20colon%20and%20rectum%20(accessed> 15 January 2021.

63. Statistics South Africa. Mortality and causes of death in South Africa: Findings from death notification. Pretoria: Statistics South Africa, 2020.

64. International Agency for Research on Cancer. Cancer Today: Population Fact sheet, South Africa. Lyon, France: IARC; 2020 [Available from: <https://gco.iarc.fr/today/data/factsheets/populations/710-south-africa-fact-sheets.pdf> accessed 02 Feb 2021.

65. Statistics South Africa. General Household Survey, 2019. Pretoria: Stats SA, 2020.

66. World Health Organisation. Musculoskeletal conditions. Geneva: World Health Organisation; 2019 [Available from: <https://www.who.int/news-room/fact-sheets/detail/musculoskeletal-conditions> accessed 18 Jan 2021.

67. World Health Organisation. Noncommunicable diseases. Geneva: WHO; 2018 [updated 1 June 2018. Available from: <https://www.who.int/en/news-room/fact-sheets/detail/noncommunicable-diseases> accessed 18 Jan 2021.

68. Koenig SM. Pulmonary complications of obesity. *The American journal of the medical sciences* 2001;321(4):249-79.

69. Zammit C, Liddicoat H, Moonsie I, et al. Obesity and respiratory diseases. *International journal of general medicine* 2010;3:335.

70. Okunogbe A, Nugent R, Spencer G, et al. Economic impacts of overweight and obesity: current and future estimates for eight countries. *BMJ Global Health* 2021;6(10)

71. Brophy T, Branson N, Daniels RC, et al. National Income Dynamics Study Panel User Manual. In: Town UoC, ed. Cape Town: South Africa, 2018.

72. Southern Africa Labour and Development Research Unit (SALDRU). Overview: National Income Dynamics Study (2008–2019), 2019.

73. Southern Africa Labour and Development Research Unit (SALDRU). National Income Dynamics Study 2017, Wave 5 [dataset]. Version 1.0.0. Cape Town: Southern Africa Labour and Development Research Unit,, 2018.

74. Global Cancer Observatory. Cancer Factsheet 2020: South Africa 2021 [Available from: <https://gco.iarc.fr/today/data/factsheets/populations/710-south-africa-fact-sheets.pdf> accessed 11 March 2021.

75. Guzha N. Development of a model to predict the cost of treatment of breast cancer with chemotherapy at Groote Schuur hospital. . University of the Western Cape 2017.

76. Feliciana Silva F, Macedo da Silva Bonfante G, Reis IA AdRH, et al. Hospitalizations and length of stay of cancer patients: A cohort study in the Brazilian Public Health System. . *PLoS ONE* 2020;15(5):e0233293. doi: <https://doi.org/10.1371/journal.pone.0233293>

77. Guh DP, Zhang W, Bansback N, et al. The incidence of co-morbidities related to obesity and overweight: a systematic review and meta-analysis. *BMC Public Pealth* 2009;9(1):1-20.

78. Gabella C. Comparison of the Costs of Treating Prostate Cancer with Standard Chemotherapy Regimens versus Targeted Nuclear Medicines. Mpharm. UKZN. UKZN, 2020.

79. Máchová L, Čížek L, Horáková D, et al. Association between obesity and cancer incidence in the population of the District Sumperk, Czech Republic. *Oncology Research and Treatment* 2007;30(11):538-42.

80. Thomsen M, Nordestgaard BG. Myocardial infarction and ischemic heart disease in overweight and obesity with and without metabolic syndrome. *JAMA internal medicine* 2014;174(1):15-22.

81. Kathard H, Padarath A, Galvaan R, et al. South African Health Review 2020. Durban: Health Systems Trust 2020.

82. Regassa L. D., Tola A. Magnitude and predictors of hospital admission, readmission, and length of stay among patients with type 2 diabetes at public hospitals of Eastern Ethiopia: a retrospective cohort study. . *BMC Endocrine Disorders* 2021;21(1):1-13.

83. Nyahoda TS. A glance at gallstones in South Africa: A one year review of sonographic findings at a tertiary hospital. University of the Witwatersrand 2016.

84. Larsson S, Wolk A. Obesity and the risk of gallbladder cancer: a meta-analysis. *British Journal of Cancer* 2007;96(9):1457-61.

85. South Africa National Department of Health. Uniform Patient Fee Schedule 2020. . Pretoia: National Department of Health, 2020.

86. National Health Laboratory Service. NHLS State Price List 2013. Johannesburg: South Africa: National Health Laboratory Service, 2013.

87. Peter JG, Theron G, Pooran A, et al. Comparison of two methods for acquisition of sputum samples for diagnosis of suspected tuberculosis in smear-negative or sputum-scarce people: a randomised controlled trial. *The Lancet Respiratory Medicine* 2013;1(6):471-78.

88. Matizirofa L, Chikobvu D. Analysing and quantifying the effect of predictors of stroke direct costs in South Africa using quantile regression. *BMC Public Health* 2021(21:1560) doi: <https://doi.org/10.1186/s12889-021-11592-0>

89. Sekeitto AR, Aden A. Costing total hip arthroplasty in a South African state tertiary hospital. *South African Medical Journal* 2021;111(3):250-54.

90. Clicks. A.Vogel Boldocynara Liver and Gall Bladder Drops 50ml 2021 [Available from: <https://clicks.co.za/a-vogel_boldocynara-liver-and-gall-bladder-drops-50ml/p/922320> accessed 10 Dec 2021.

91. Herbst C-l, Miot JK, Moch SL, et al. Access to colorectal cancer (crc) chemotherapy and the associated costs in a south african public healthcare patient cohort. *Journal of Cancer Policy* 2018;15:18-24.

92. South Africa National Department of Health. Clinical guidelines for breast cancer control and management. Pretoria: National Department of Health, 2018.

93. Anderson D, Barnes R, Bida Meshack, et al. South African Prostate Cancer Guidelines - Draft 2017: South African Urological Association, 2017.

94. Parker C, Gillessen S, Heidenrich A, et al. Cancer of the prostate: ESMO Clinical Practice Guidelines for diagnosis, treatment and follow up. *Annals of Oncology* 2020;31(9):1119-34.

95. Cancer Therapy Advisor. Breast Cancer (Invasive; Nonmetastatic) Treatment Regimens. 2020 [Available from: <https://www.cancertherapyadvisor.com/home/cancer-topics/breast-cancer/breast-cancer-treatment-regimens/breast-cancer-invasive-treatment-regimens/> accessed 01 April 2021.

96. Candice-lee H. Cost Analysis of Colorectal Cancer Chemotherapy Treatment in Public and Private Healthcare Sectors in South Africa. University of the Witwatersrand, 2017.

97. Government of South Africa. Contract circular: contract description: supply and delivery of oncology and immunological agents to the state for the period 1 July 2018 to 30 June 2020. Pretoria: Government of South Africa, 2018.

98. Government of South Africa. Contract circular with NSN: supply and delivery of solid dosage forms to the state for the period 1 May 2019 t0 30 April 2021. Pretoria: South Africa: Government of South Africa, 2019.

99. Wholesale Surgical. Wintergreen 2020 [Available from: <https://www.wholesurgical.co.za/transpharm-90263-oil-of-wintergreen-500ml-pharmachemp?gclid=Cj0KCQiA8vSOBhCkARIsAGdp6RSQitTuoxcdNuBYjvGuO3fZ0GgKxVN-mWuc-6V6PbwbK7Reu5OstncaAjB3EALw_wcB> accessed April 08 2021.

100. Travel Doctor. Travel doctor Johannesburg price list [Available from: <https://traveldoctor.co.za/price-list/> accessed April 15 2021.

101. South African National Department of Health. Adult Primary Care 2018/19. Pretoria: National Department of Health, 2019.

102. Anthea Rhoda, Natalie Cunningham, Simon Azaria, et al. Provision of inpatient rehabilitation and challenges experienced with participation post discharge: quantitative and qualitative inquiry of African stroke patients. *BMC Health Services Research* 2015;15 doi: <https://doi.org/10.1186/s12913-015-1057-z>

Table 5: Characteristics of the studies included in the review.

| Title/Study | Publication Year | Setting | Income classification | Time Horizon | Source |
| --- | --- | --- | --- | --- | --- |
| The projected burden of primary total knee and hip replacement for osteoarthritis in Australia to the year 2030." | 2019 | Australia | High Income | 2013-2030 | ^14^ |
| "Current and future costs of cancer, heart disease and stroke attributable to obesity in Australia - a comparison of two birth cohorts." | 2009 | Australia | High Income | >1 year | ^15^ |
| Obesity and overweight in Canada: an updated cost-of-illness study. | 2010 | Canada | High Income | 1 year | ^17^ |
| The costs of overweight and obesity-related diseases in the Brazilian public health system: cross-sectional study. | 2012 | Brazil | upper middle | 3 year average | ^21^ |
| The cost of obesity in Canada. | 1999 | Canada | High Income | 1 year | ^18^ |
| Economic costs of obesity. | 1992 | United States | High Income | 1 year | ^28^ |
| Direct healthcare cost of obesity in Brazil: an application of the cost-of-illness method from the perspective of the public health system in 2011. | 2015 | Brazil | upper middle | 1 year | ^22^ |
| Costs of metabolic syndrome-related diseases induced by obesity in Taiwan. | 2008 | Taiwan | High Income | 1 year | ^36^ |
| Burden of Disease Attributable to Obesity and Overweight in Colombia. | 2019 | Columbia | upper middle | 1 year | ^37^ |
| Excess Costs and Economic Burden of Obesity-Related Cancers in the United States. | 2019 | United States | High Income | 13 years | ^25^ |
| Health care cost of overweight-related diseases in Bangladesh. | 2020 | Bangladesh | lower middle | 1 year | ^38^ |
| Socioeconomic costs of overweight and obesity in Korean adults. | 2011 | South Korea | High Income | 1 year | ^39^ |
| Application of the UK foresight obesity model in Ireland: the health and economic consequences of projected obesity trends in Ireland. | 2013 | Ireland | High Income | 2010-2030 | ^42^ |
| Health burden and costs of obesity and overweight in Germany. | 2011 | Germany | High Income | 1 year | ^44^ |
| Variation across Canada in the economic burden attributable to excess weight, tobacco smoking and physical inactivity. | 2015 | Canada | High Income | Annual (1 year) | ^19^ |
| Health care and lost productivity costs of overweight and obesity in New Zealand. | 2012 | New Zealand | High Income | 1 year | ^47^ |
| The economic burden of cancer attributable to obesity in Korea: A population-based cohort study. | 2019 | South Korea | High Income | 2002-2015 | ^40^ |
| Trends in socioeconomic costs of morbid obesity among Korean adults, 2009-2013: Data from National Health Insurance Service. | 2018 | South Korea | High Income | 2009-2013 | ^41^ |
| Health Burden and Costs of Obesity and Overweight in Germany: An Update. | 2015 | Germany | High Income | 2 year | ^45^ |
| Health Care Costs Attributable to Overweight Calculated in a Standardized Way for Three European Countries. | 2016 | Netherlands, Germany, Czech Republic | High Income | 1 year | ^59^ |
| Economic burden of obesity and four obesity-related chronic diseases in rural Yunnan Province, China. | 2018 | China -provincial level | upper middle | 1 year | ^49^ |
| Forecasting the future economic burden of current adolescent overweight: an estimate of the coronary heart disease policy model. | 2009 | United States | High Income | 30 years | ^29^ |
| Hospitalization rates and cost in severe or complicated obesity: an Italian cohort study. | 2013 | Italy | High Income | >1 year | ^53^ |
| The cost of obesity and overweight in 2005: a case study of Alberta, Canada. | 2011 | Canada, Alberta | High Income | 1 year | ^20^ |
| The clinical and economic burden of obesity in a managed care setting. | 2000 | United States | High Income | 1 year | ^30^ |
| The economic burden of preventable chronic diseases in Rhode Island. | 2014 | United States | High Income | 1 year | ^31^ |
| Economic costs of obesity in Thailand: a retrospective cost-of-illness study. | 2014 | Thailand | upper middle | 1 year | ^54^ |
| Measuring the full economic costs of diet, physical activity and obesity-related chronic diseases. | 2006 | China | upper middle | 1 year | ^52^ |
| Estimating Premature Mortality Cost of Cancers Attributable to Obesity in Indonesia. | 2019 | Indonesia | upper middle | 1 year | ^55^ |
| Obesity prevalence in Mexico: impact on health and economic burden. | 2014 | Mexico | upper middle | 40 year simulation | ^56^ |
| Health and economic burden of obesity in Brazil. | 2013 | Brazil | upper middle | 40 year simulation | ^23^ |
| Economic Burden of Obesity and Its Complications in Germany. | 2003 | Germany | high income | 1 year | ^46^ |
| The economic burden of ill health due to diet, physical inactivity, smoking, alcohol and obesity in the UK: an update to 2006-07 NHS costs. | 2011 | UK | High Income | 1 year | ^57^ |
| Economic burden of obesity and its comorbidities in Switzerland. | 2005 | Switzerland | High Income | 1 year | ^58^ |
| The cost of obesity: the Australian perspective. | 1994 | Australia | High Income | 1 year | ^16^ |
| The burden of hospitalization due to overweight and obesity in Brazil. | 2007 | Brazil | upper middle | 1 year | ^24^ |
| Health care costs of obesity in New Zealand. | 1997 | New Zealand | High Income | 1 year | ^48^ |
| Estimated economic costs of obesity to U.S. business. | 1998 | United States | High Income | 1 year | ^32^ |
| The cost of obesity - the United-States perspective. | 1994 | United States | High Income | 1 year | ^33^ |
| Social and economic effects of body weight in the United States. | 1996 | United States | High Income | 1 year | ^26^ |
| Current estimates of the economic cost of obesity in the United States. | 1998 | United States | High Income | 1 year | ^27^ |
| Economic burden of obesity-related chronic diseases in Mainland China. | 2008 | China | upper middle | 1 year | ^51^ |
| The economic cost of physical inactivity in China. | 2013 | China | upper middle | 1 year | ^50^ |
| Annual Burden and Costs of Hospitalization for High-Need, High-Cost Patients with Chronic Gastrointestinal and Liver Diseases. | 2018 | United States | High Income | 1 year | ^34^ |
| Economic burden of cardiovascular disease associated with excess body weight in U.S. adults. | 2002 | United States | High Income | >1 year | ^35^ |

Table 6: Costing Methods and types of cost included in reviewed studies.

| Title/Study | Publication Year | Direct Cost Method | Indirect Cost Method | | Types of Cost | |  | Source |
| --- | --- | --- | --- | --- | --- | --- | --- | --- |
|  |  |  |  | | DHC | DNHC | IC |  |
| The projected burden of primary total knee and hip replacement for osteoarthritis in Australia to the year 2030." | 2019 | Bottom up, prevalence-based attributable fractions | N/A | | ✓ | - | - | ^14^ |
| "Current and future costs of cancer, heart disease and stroke attributable to obesity in Australia - a comparison of two birth cohorts." | 2009 | Bottom up, prevalence-based attributable fractions | N/A | | ✓ |  | - | ^15^ |
| Obesity and overweight in Canada: an updated cost-of-illness study. | 2010 | prevalence-based, top- down | human capital approach | | ✓ | - | ✓ | ^17^ |
| The costs of overweight and obesity-related diseases in the Brazilian public health system: cross-sectional study. | 2012 | Bottom up | N/A | | ✓ | - |  | ^21^ |
| The cost of obesity in Canada. | 1999 | top down | N/A | | ✓ | - | - | ^18^ |
| Economic costs of obesity. | 1992 | prevalence-based, top down | human capital based | | ✓ |  | ✓ | ^28^ |
| Direct healthcare cost of obesity in brazil: an application of the cost-of-illness method from the perspective of the public health system in 2011. | 2015 | top down, attributable fraction | N/A | | ✓ | - | - | ^22^ |
| Costs of metabolic syndrome-related diseases induced by obesity in Taiwan. | 2008 | top down | N/A | | ✓ | - | - | ^36^ |
| Burden of Disease Attributable to Obesity and Overweight in Colombia. | 2019 | top down approach | N/A | | ✓ | - | - | ^37^ |
| Excess Costs and Economic Burden of Obesity-Related Cancers in the United States. | 2019 | survey data; regression | N/A | | ✓ | - | - | ^25^ |
| Health care cost of overweight-related diseases in Bangladesh. | 2020 | survey data, bottom up, attributable fractions | N/A | | ✓ |  |  | ^38^ |
| Socioeconomic costs of overweight and obesity in Korean adults. | 2011 | top down | human capital approach | | ✓ | - | ✓ | ^39^ |
| Application of the UK foresight obesity model in Ireland: the health and economic consequences of projected obesity trends in Ireland. | 2013 | microsimulation and regression | N/A | | ✓ |  | - | ^42^ |
| Health burden and costs of obesity and overweight in Germany. | 2011 | top down approach | human capital approach | | ✓ | - | ✓ | ^44^ |
| Variation across Canada in the economic burden attributable to excess weight, tobacco smoking and physical inactivity. | 2015 | top down | human capital approach: based on average annual earnings and employment rates | | ✓ | - | ✓ | ^19^ |
| Health care and lost productivity costs of overweight and obesity in New Zealand. | 2012 | bottom-up; top down approach, attributable fractions | human capital and friction cost approach | | ✓ | - | ✓ | ^47^ |
| The economic burden of cancer attributable to obesity in Korea: A population-based cohort study. | 2019 | bottom up; attributable fractions based on cohort study | human capital | | ✓ | ✓ | ✓ | ^40^ |
| Trends in socioeconomic costs of morbid obesity among Korean adults, 2009-2013: Data from National Health Insurance Service. | 2018 | top down, attributable fractions | human capital | | ✓ | - | ✓ | ^41^ |
| Health Burden and Costs of Obesity and Overweight in Germany: An Update. | 2015 | top down approach | human capital approach; friction | ✓ | | - | ✓ | ^45^ |
| Health Care Costs Attributable to Overweight Calculated in a Standardized Way for Three European Countries. | 2016 | top down approach, prevalence-based | N/A | | ✓ | - | - | ^59^ |
| Economic burden of obesity and four obesity-related chronic diseases in rural Yunnan Province, China. | 2018 | top down | human capital | | ✓ | - | - | ^49^ |
| Forecasting the future economic burden of current adolescent overweight: an estimate of the coronary heart disease policy model. | 2009 | top down approach, microsimulation | human capital | | ✓ | - | - | ^29^ |
| Hospitalization rates and cost in severe or complicated obesity: an Italian cohort study. | 2013 | retrospective cohort study; bottom up | N/A | | ✓ | - | - | ^53^ |
| The cost of obesity and overweight in 2005: a case study of Alberta, Canada. | 2011 | top down | Human capital approach | | ✓ |  | ✓ | ^20^ |
| The clinical and economic burden of obesity in a managed care setting. | 2000 | survey, top down approach | N/A | | ✓ | - | - | ^30^ |
| The economic burden of preventable chronic diseases in Rhode Island. | 2014 | bottom up approach | human capital | | ✓ |  | ✓ | ^31^ |
| Economic costs of obesity in Thailand: a retrospective cost-of-illness study. | 2014 | bottom up; retrospective study | human capital | | ✓ |  | ✓ | ^54^ |
| Measuring the full economic costs of diet, physical activity and obesity-related chronic diseases. | 2006 | top down approach; attributable fractions | unclear, description suggests human capital | | ✓ | - | ✓ | ^52^ |
| Estimating Premature Mortality Cost of Cancers Attributable to Obesity in Indonesia. | 2019 | human capital approach: using average monthly income | productivity losses: premature mortality cost | | - | - | ✓ | ^55^ |
| Obesity prevalence in Mexico: impact on health and economic burden. | 2014 | uses cross-sectional data; regression; top down | N/A | | ✓ | - | - | ^56^ |
| Health and economic burden of obesity in Brazil. | 2013 | uses cross-sectional data; regression, top down | N/A | | ✓ | - | - | ^23^ |
| Economic Burden of Obesity and Its Complications in Germany. | 2003 | top down approach, attributable fraction | human capital approach | | ✓ | - | ✓ | ^46^ |
| The economic burden of ill health due to diet, physical inactivity, smoking, alcohol and obesity in the UK: an update to 2006-07 NHS costs. | 2011 | top down, attributable fractions | N/A | | ✓ |  | - | ^57^ |
| Economic burden of obesity and its comorbidities in Switzerland. | 2005 | bottom up approach | human capital | | ✓ | - | ✓ | ^58^ |
| The cost of obesity: the Australian perspective. | 1994 | bottom up approach; attributable fractions | N/A | | ✓ | - | - | ^16^ |
| The burden of hospitalization due to overweight and obesity in Brazil. | 2007 | survey analysis: retrospective study and attributable fraction | N/A | | ✓ | - | - | ^24^ |
| Health care costs of obesity in New Zealand. | 1997 | bottom up | N/A | | ✓ | - | - | ^48^ |
| Estimated economic costs of obesity to U.S. business. | 1998 | top-down | human capital approach | | ✓ | - | ✓ | ^32^ |
| THE COST OF OBESITY - THE UNITED-STATES PERSPECTIVE. | 1994 | top-down | human capital | | ✓ | - | ✓ | ^33^ |
| Social and economic effects of body weight in the United States. | 1996 | top down | regression analysis of survey data | | ✓ | - | ✓ | ^26^ |
| Current estimates of the economic cost of obesity in the United States. | 1998 | bottom up; top down | human capital | | ✓ | - | ✓ | ^27^ |
| Economic burden of obesity-related chronic diseases in Mainland China. | 2008 | cohort study, bottom-up, attributable fractions | N/A | | ✓ | - | - | ^51^ |
| The economic cost of physical inactivity in China. | 2013 | survey data; attributable fractions | human capital | | ✓ | - | - | ^50^ |
| Annual Burden and Costs of Hospitalization for High-Need, High-Cost Patients With Chronic Gastrointestinal and Liver Diseases. | 2018 | bottom-up | N/A | | ✓ | - | - | ^34^ |
| Economic burden of cardiovascular disease associated with excess body weight in U.S. adults. | 2002 | survey, Bottom-up | N/A | | ✓ | - | - | ^35^ |

✓ included; - not included; DHC: Direct healthcare cost; DNHC: direct nonhealthcare cost; N/A: not applicable
